# Zeolites ameliorate asbestos toxicity in a transgenic model of malignant mesothelioma

**DOI:** 10.1096/fba.2019-00040

**Published:** 2019-08-22

**Authors:** Xiyong Fan, Chris McLaughlin, Cleo Robinson, Jason Ravasini, Karin Schelch, Thomas Johnson, Nico van Zandwijk, Glen Reid, Anthony M. George

**Affiliations:** ^1^ School of Life Sciences University of Technology Sydney Broadway NSW Australia; ^2^ School of Biomedical Sciences University of Western Australia (M503) Crawley WA Australia; ^3^ Molecular Anatomical Pathology, PathWest Laboratory Medicine QEII Medical Centre Nedlands WA Australia; ^4^ Asbestos Diseases Research Institute University of Sydney Sydney NSW Australia; ^5^ Faculty of Medicine University of Sydney Sydney NSW Australia; ^6^ Institute of Cancer Research, Department of Medicine I Medical University of Vienna Vienna Austria; ^7^ Present address: Department of Pathology University of Otago Dunedin New Zealand

**Keywords:** asbestos, Mesothelioma, mouse model, zeolites

## Abstract

Malignant mesothelioma (MM) is an almost invariably fatal cancer caused by asbestos exposure. The toxicity of asbestos fibers is related to their physicochemical properties and the generation of free radicals. We set up a pilot study to investigate the potential of the zeolite clinoptilolite to counteract the asbestos carcinogenesis by preventing the generation of reactive nitrogen and oxygen radicals. In cell culture experiments, clinoptilolite prevented asbestos‐induced cell death, reactive oxygen species production, DNA degradation, and overexpression of genes known to be up‐regulated by asbestos. In an asbestos‐induced transgenic mouse model of MM, mice were injected intraperitoneal injections with blue asbestos, with or without clinoptilolite, and monitored for 30 weeks. By the end of the trial all 13 mice injected with asbestos alone had reached humane end points, whereas only 7 of 29 mice receiving crocidolite and clinoptilolite reached a similar stage of disease. Post‐mortem examination revealed pinpoint mesothelioma‐like tumors in affected mice, and the absence of tumor formation in surviving mice. Interestingly, the macrophage clearance system, which was largely suppressed in asbestos‐treated mice, exhibited evidence of increased phagocytosis in mice treated with asbestos and clinoptilolite. Our study suggests that inhibiting the asbestos‐induced generation of reactive oxygen species and stimulating the macrophage system may represent a pathway to amelioration of asbestos‐induced toxicity. Additional studies are warranted to explore the underlying mechanisms responsible for our observations.

## 1 INTRODUCTION

Asbestos is a naturally occurring fibrous silicate mineral that was a popular building material until the late 20th century. Asbestos can be classified into two main families: the serpentines, consisting of chrysotile (white asbestos) and amphiboles which includes crocidolite (blue asbestos), tremolite and amosite.^1^, ^2^ White asbestos (chrysotile) was specifically and widely used in asbestos cement as a building material. It was also used, along with other types of asbestos in other industrial materials, and in automobile brake linings and as insulation for pipes, ducts, electrical goods, and extensively in ship building. In all, asbestos was used in more than 3000 products.^1^ Although asbestos has been recognized as a human carcinogen since 1955,^3^ the general public only became aware of its profound dangers in the 1980s. Despite this, its use has been banned in only 66/195 countries, as of March 2019. Asbestos‐related cancers include lung cancer and malignant mesothelioma (MM).^4^, ^5^ MM arises in the mesothelial membrane lining the pleura and peritoneum and is considered a poor‐prognosis cancer.^6^ There is no cure for MM and most patients die within 2 years of diagnosis. Best practice palliative chemotherapy yields a mean survival improvement of around 3 months.^7^, ^8^ The incidence of MM is still rising in most industrialized countries and will increase in developing countries due to continued use of asbestos.^9^

Multiple studies suggest that iron present in, or adsorbed to, asbestos fibers is a key factor for asbestos toxicity and the formation of asbestos bodies in the lung that are the hallmarks of asbestos exposure.^2^, ^10^, ^11^ Iron from asbestos fibers (released as labile Fe^3+^) catalyzes the generation of reactive oxygen and nitrogen species, induces DNA single strand breaks, lipid peroxidation, and the release of proinflammatory cytokines, leading to genotoxicity, reduced immune responsiveness and, eventually, MM.^12^, ^13^, ^14^, ^15^, ^16^, ^17^, ^18^ The mechanisms leading to the development of asbestos‐related cancers are complex and poorly understood.

Asbestos‐induced and genetically modified mouse models have been developed in order to better understand asbestos carcinogenicity in MM. One example is the MexTAg299 transgenic mouse model, in which the Simian Virus (SV40) large T antigen is expressed specifically in mesothelial cells under the control of the mesothelial cell‐specific mesothelin promoter.^19^ When exposed to asbestos, these mice develop cancers at 100% incidence within 40 weeks, and with tumors that replicate key features of the pathogenesis of human MM.^20^

Zeolites are a group of hydrated natural or synthetic microporous crystals containing AlO_4_ and SiO_4_ tetrahedra, linked through common oxygen atoms into rigid anionic cage frameworks containing well‐defined channels and cavities, which give zeolites a high capacity for ion exchange.^21^ The majority of natural zeolites are of volcanic origin and have the general formula, M2/*n*:Al_2_O_3_:*x*SiO_2_:*y*H_2_O, where M stands for the extra‐framework cation. A recent review summarizes the origins, structures and properties, and industrial and medical and veterinary uses of natural and synthetic zeolites.^22^ An emphasis on the most commonly used zeolite in humans, namely clinoptilolite, is combined with hypotheses to explain observed positive effects of detoxification, immune response, and general health in humans.

Naturally occurring zeolites include those whose biological and chemical reactivity is reportedly carcinogenic (erionite) to essentially non‐toxic (clinoptilolite). Erionite's carcinogenic propensity derives from its asbestos‐like fibrous structure.^23^, ^24^, ^25^ Erionite‐induced mesotheliomas are similar to those originating from asbestos in exhibiting biopersistence, long latency, histology, and fibrosis. Erionite is listed on the American Cancer Society website as a risk factor for MM, along with asbestos, SV40, and radiation. Most other zeolites such as clinoptilolite, mordenite, and chabazite have non‐toxic, less reactive orthorhombic crystalline structures, with accessible open channels for “capturing” or sequestering water and large ions.^21^, ^26^

The present study began with the notion that the metal‐binding properties of zeolites would scavenge iron released from asbestos fibers and consequently ameliorate asbestos toxicity. Here we demonstrate the moderating effects of clinoptilolite on asbestos‐induced cellular damage in vitro and asbestos‐induced carcinogenesis in vivo. We discuss the potential implications of these findings and the future studies needed to understand the mechanisms underlying the asbestos ameliorating effects of clinoptilolite.

## 2 METHODS

### 2.1 Preparation of asbestos fibers

White (chrysotile) and blue (crocidolite) asbestos fibers were obtained from two sources: The Union for International Cancer Control (UICC; Switzerland); and James Hardie Industries (Australia). Fiberglass was sourced from Bradford Insulation (Sydney, Australia). Fibers in methanol were processed in a Fritsch pulverisette23 mini‐mill (Fritsch GmbH, Germany) to a uniform size, which was determined by a laser particle scanner to average dimensions of 10 µm in length by 1 µm width. Fiber suspensions were prepared in phosphate‐buffered saline (PBS) at a pH of 7.4 and stored at 4‐6°C. Crocidolite and chrysotile asbestos fibers were diluted to concentrations of 10 and 5 mg/mL, respectively, on the day of the experiment. Fiberglass was pulverized in a mortar and pestle to a fine dust, then suspended in PBS at 10 mg/mL and refrigerated. For cell culture experiments, fibers were washed twice in cell culture medium without fetal bovine serum (FBS) and resuspended in culture medium (without FBS) to 1 mg/mL.

### 2.2 Zeolites used in this study

Natural clinoptilolite (NCL) was obtained from St. Cloud. Synthetic clinoptilolite (SCL), and synthetic mordenite (SMO) were obtained from Novachem. NCL was chosen because it is one of the most common and well described zeolites. The two synthetic zeolites were chosen to give a comparison with NCL in the cell culture experiments. For the mouse model trials, only NCL was used. Zeolites were ground in a Fritsch mill, scanned, and stored in PBS. The milled mean NCL particle size was 4.28 µm for NCL. For some assays, SCL and SMO were tested alongside NCL. Their milled mean particle sizes were 4.73 and 5.26 µm, respectively.

### 2.3 Cell culture and treatment with fibers or minerals

A549 and MeT‐5A cells were obtained from the American Type Culture Collection (ATCC) and stored in liquid nitrogen at a density of 1 × 10^6^ cells/mL in 1 mL cryogenic storage vials. Cells cultures were grown in complete medium (RPMI1640; Life Technologies), supplemented with 10% FBS, and 100 units/mL penicillin and streptomycin, at 37°C with 5% CO_2_. Cells were treated with fiberglass, asbestos, or zeolites as required in T25 or T75 flasks or 6‐ 24‐ or 96‐well plates. Medium containing asbestos, fiberglass, or zeolites was added at concentrations of, 0.5, 1, 2.5, 5, or 10 µg/cm^2^, and incubation continued for 24‐72 h. Cells were then analyzed for viability, ROS production, DNA fragmentation, or gene expression as outlined below.

### 2.4 Cell viability assay

When cell confluence reached 70%‐80% in six‐well plates, they were treated with blue (crocidolite) or white (chrysotile) asbestos (at 5 µg/cm^2^) with or without natural or SCL, or mordenite (all at 10 µg/cm^2^) for 24‐36 hours, then trypsinized and washed three times in PBS and assayed for viability using propidium iodide and scored in a Tali Image‐Based Cytometer at nine fields per sample. Each sample was derived from pooled cells from a six‐well plate and each assay was repeated three times.

### 2.5 Reactive oxygen species assay

A549 or MeT‐5A cells were cultured to 70%‐80% confluence in six‐well plates, treated with asbestos and/or zeolites for 24‐36 hours, as in the viability assays, then treated with CellROX Orange (Life Technologies) using the supplier's protocol, followed by measurement of ROS fluorescence in a Tali Image‐Based Cytometer using the same number of fields and pooled cells as in the viability assays. CellROX reagents are fluorogenic probes for measuring generalized oxidative stress in cells, and were used here to measure levels of ROS in response to different treatments. While reagents selective for a variety of reactive species targeted to the cytosol or mitochondria are available, these were considered beyond the scope of the current study.

### 2.6 DNA extraction and gel electrophoresis

A549 cells were seeded at a density of 5.0 × 10^4^ cells/mL in T75 flasks and incubated for 24 hours, then treated with asbestos, fiberglass, or NCL and incubated for a further 72 hours. Confluent cells were harvested by trypsinisation, centrifuged, and washed in PBS. DNA extraction from PBS resuspended cell pellets was performed using Trizol reagent (Invitrogen) and the manufacturer's protocol. Final DNA pellets were resuspended in 200 µL of 8 mmol/L NaOH and quantified in a Nanodrop spectrophotometer. DNA samples of 150 ng/µL (~10 µL per well) were run in 1.2% agarose gels in TBE buffer (with Gel‐Red), and photographed in UV light.

### 2.7 Gene expression analysis with RT‐qPCR

We selected two genes (EGR1; IL‐8) known to be induced by exposure of A549 cells to crocidolite 27 and used RT‐qPCR to monitor changes in gene expression following challenge with crocidolite, chrysotile, and NCL. Initially, cells were titrated with 1‐10 µg/cm^2^ asbestos and changes in expression were observed in a dose‐dependent response. Concentrations of 5 µg/cm^2^ crocidolite or chrysotile and 10 µg/cm^2^ NCL were chosen as test concentrations. A549 cells (1 × 10^6^ cells in 5 mL complete medium) were seeded into flasks and incubated to achieve 70% confluence at the time of exposure to asbestos or NCL. Fresh medium containing asbestos or asbestos and NCL was added and growth continued for 24 hours, then cells were washed in PBS. RNA was isolated using Trizol (Invitrogen) following the manufacturer's protocol. Final RNA pellets were resuspended in RNase‐free water and quantified by Nanodrop at 260/230 nm. Reverse transcription was performed on total RNA (2 µg) using the High Capacity cDNA reverse transcription kit (Applied Biosystems) according to the supplier's instructions. Comparative RT‐qPCR was performed on cDNA using TaqMan Gene Expression Master Mix (Applied Biosystems) and EGR1‐ and IL‐8‐specific primers, with 18S RNA used as reference to estimate fold‐changes in EGR1 or IL‐8 expression.

### 2.8 Transgenic mice and asbestos‐induced MM trial

The trials were conducted essentially as described,^20^ with mixed‐sex MexTAg299h transgenic mice at 17‐19 weeks age. They were fed a basal diet and given water ad libitum. Intraperitoneal injections (i.p.) were given as follows: Control subgroups of three mice were injected with normal saline or with NCL (90 mg/kg) weekly. Twelve mice were given crocidolite (120 mg/kg; approx. 3 mg per injection; weeks 1 and 4). Eighteen mice were given crocidolite (weeks 1 and 4) plus NCL (30 or 90 mg/kg) weekly. Six mice were given crocidolite (weeks 1 and 4) plus NCL (30 mg/kg) weekly until week 10). Six mice were given crocidolite (weeks 1 and 4) then NCL (30 mg/kg) weekly from week 10. This protocol of injections was designed to test the ability of NCL given at the same time as asbestos to delay or prevent the onset of MM; given at the same time then withdrawn to test the time span of any protective effect of NCL; or given 10 weeks after the first asbestos injection to test NCL as a moderator of the process of MM tumor development that may have already begun. Complete post‐mortem data for the two trials are given in Tables S1 and S2. Asbestos fibers and NCL were prepared in saline on the days of the injections. Mice were monitored for general well‐being and weighed daily until humane endpoints were reached, defined as rapid weight loss or gain, distension of the gut, hunching of the back, and signs of sickness, distress, or loss of mobility. Survival was taken from the date of the first injection. Mice were terminated by CO_2_ asphyxia. Mouse trials had Institutional Biosafety and Animal Care & Ethics Committees clearances and were carried out according to standard operating protocols.

### 2.9 Histology

Tissue samples were fixed in 4% paraformaldehyde in PBS for 24 hours and preserved in 70% ethanol until processed into paraffin blocks. Five micrometer sections were cut from paraffin blocks, dewaxed in xylene and rehydrated in a graded series of alcohols. The dewaxed sections were then subjected to either haematoxylin and eosin staining or Perl's iron staining. Crocidolite fibers were counted under 40× magnification after Perl's iron staining. For each section, 10 consecutive fields along the peritoneum were chosen to count intact fibers, and another 10 consecutive tissue fields adjacent to the peritoneum were chosen to count phagocytotic fibers. For each mouse, 10 consecutive sections on the same levels were cut, and three of the 10 sections were chosen randomly for fiber counts. The averaged total numbers of the fibers in each set of 10 fields (at 40×) were recorded and plotted. All mice were sectioned as above, with three from each subgroup chosen at random for the counts.

### 2.10 Immunohistochemistry

Immunohistochemistry was performed by modifications of several methods.^20^, ^28^, ^29^ The SV40 pAb 101 antibody (Santa Cruz Biotechnology) was applied to the deparaffinized sections at 1:100 dilution at 4°C in a moist chamber overnight; and detected using a biotinylated horse anti‐mouse IgG (Vector Laboratories) with the Avidin‐Biotin‐Enzyme Complex (Vector Laboratories) and diaminobenzamidine (Sigma‐Aldrich) as described previously.^30^ Samples were counter‐stained in Mayer's hematoxylin before embedding in DEPEX. SV‐40 positive mesothelial cells and Kupffer cells were counted under 40× magnification after immune staining. For each section, 10 consecutive fields along the peritoneum were chosen to count SV‐40 positive mesothelial cells, and another ten consecutive tissue fields adjacent to the peritoneum were chosen to count SV‐40 positive Kupffer cells. For each animal, three of the 10 consecutive sections on the same level were chosen randomly for cell counts. The averaged total numbers of cells in each set of 10 fields were recorded as the final result.

### 2.11 Statistical analyses

Statistical analysis was performed using spss 19.0 and Graphpad Prism 6.02 software. Kaplan Meier survival curves (Fig. 5) were calculated and plotted using Graphpad Prism. Cell culture experiments were performed in triplicate and repeated two to four times. Data in the figures of the mouse trial are provided as means ± SEM, where *P* < .05 was considered significant. Statistical comparisons in cell culture experiments were performed using one‐way ANOVA with Bonferroni post hoc test to analyze group differences, and the probability of *P* < .05 was considered significant. The two‐way ANOVA with Sidak's post‐test was used to analyze group differences in the mouse trial for organ cell counts from histology and immunohistochemistry slides. The size of the mouse subgroups (n = 6) was determined by statistical analysis using GPower 3.1 analysis (http://www.gpower.hhu.de/en.htm), assuming a *z*‐test to compare proportions at a significance level of 0.05. A sample size of six mice in each treatment subgroup was sufficient to provide over 80% power to detect the difference between these groups using a two‐tailed test, with the exception of the control group in Trial 1 which had only three mice.

## 3 RESULTS

### 3.1 Zeolites moderate the effects of asbestos fibers in human lung cell lines

**Figure 1:**
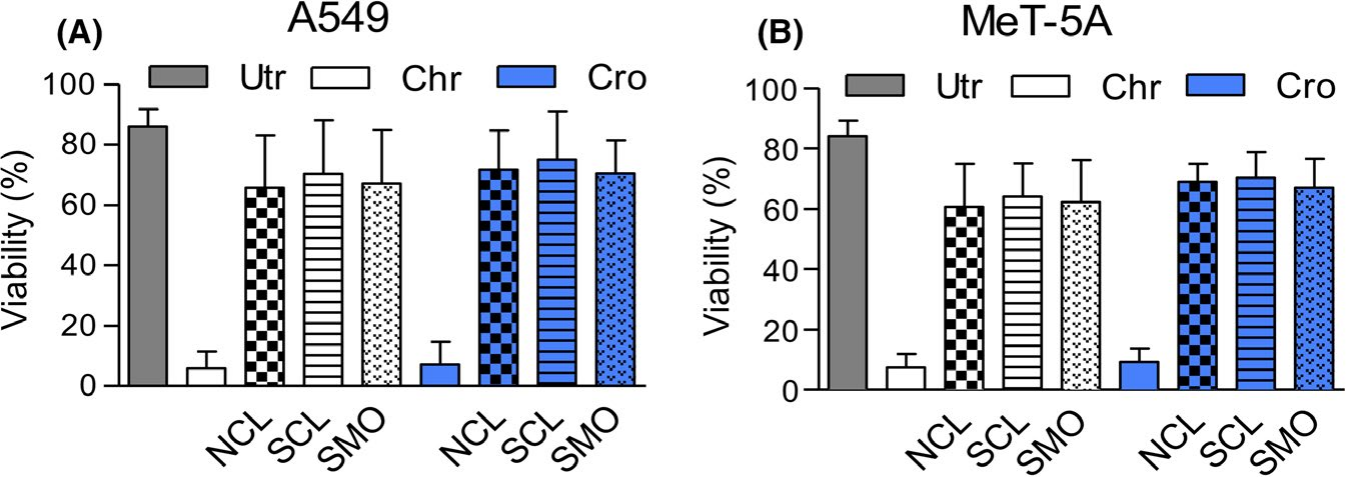
Zeolites ameliorate toxic effects of asbestos fibers in vitro. Viability of A549 (A) and MeT‐5A (B) cells subjected to various treatments. Untreated cells (Utr) and those treated with chrysotile (Chr) or crocidolite (Cro) are as labeled. Effects of asbestos were reduced in cells additionally treated with natural clinoptilolite (NCL), synthetic clinoptilolite (SCL), or synthetic mordenite (SMO). Data were pooled from triplicate experiments repeated three times; and the percentage survival normalized to untreated cells. Crocidolite or chrysotile was added at 5 µg/cm^2^ with or without natural or synthetic clinoptilolite, or mordenite (all at 10 µg/cm^2^) for 24‐36 hr Viability plots for zeolites alone were not shown in this figure as the survival levels were near identical (within a few percent) of untreated cells (Utr). However, viability plots for all three zeolites are given in Fig. S1

In both lung adenocarcinoma (A549) and mesothelial (MeT‐5A) cell lines, asbestos‐induced cellular toxicity, increase in ROS, genomic DNA degradation, and overexpression of genes up‐regulated by asbestos, were ameliorated by co‐administered zeolites, namely NCL, SCL, or SMO. The origins and uses of these three zeolites and the reasons for using them in this study is discussed at the beginning of Section 2. The surviving fraction of cells was <10% after chrysotile or crocidolite exposure but increased significantly (>60%; *P* < .0001) when NCL, SCL, or SMO were included (Fig. 1). A549 or MeT‐5A cells treated with each of the three zeolites alone, at up to 10 times the level used in the viability assay, showed them to be essentially non‐toxic (Fig. S1).

**Figure 2:**
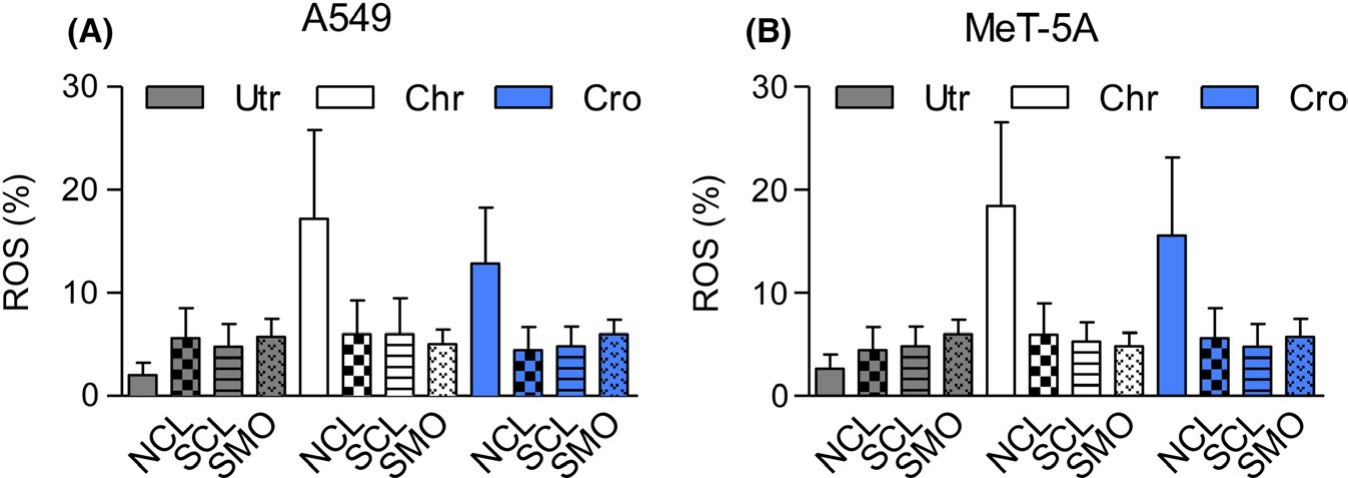
Reactive oxygen species (ROS), detected as increase in fluorescence, was measured in a Tali Cytometer. Percentage ROS was expressed as a percentage of stained/unstained cells and plotted for A549 (A) and MeT‐5A (B) cells subjected to various treatments. Legend as in Fig. 1. Crocidolite or chrysotile was added at 5 µg/cm^2^ with or without natural or synthetic clinoptilolite, or mordenite (all at 10 µg/cm^2^) for 24‐36 h

The considerable increase in ROS levels in A549 and MeT‐5A cells after asbestos exposure was prevented by the simultaneous presence of zeolites (Fig. 2). An increase of ROS in asbestos‐treated cells and downstream stress responses are well‐documented,^14^, ^31^, ^32^ as is its reversal with iron chelating agents or hydroxyl radical scavengers.^33^ The high iron content of asbestos appears to be critical to the genesis of ROS, which is chiefly manifested as the highly reactive hydroxyl radical (HO˙) from H_2_O_2_ with oxidation of ferrous iron (Fe^2+^) to ferric iron (Fe^3+^) via the Fenton reaction.^24^

**Figure 3:**
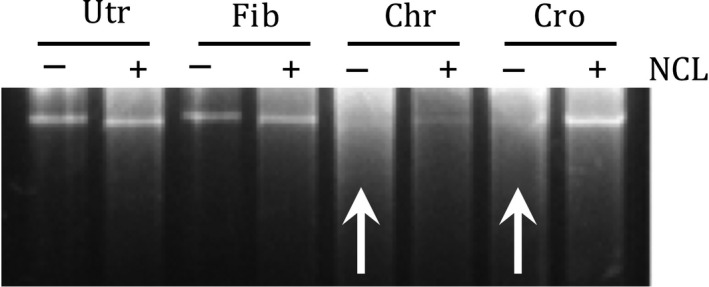
Agarose gel electrophoretic analysis of genomic DNA from A549 cells after 72 h treatment with fiberglass (Fib), chrysotile (Chr), or crocidolite (Cro), all at 5 µg/cm^2^, with or without natural clinoptilolite (NCL) at 10 µg/cm^2^. Upward facing white arrows indicate the degraded DNA lanes for asbestos alone

Genomic DNA from A459 cells was heavily degraded in cells exposed to chrysotile or crocidolite fibers, but largely intact in cells exposed to asbestos plus NCL (Fig. 3). DNA was not affected by NCL alone or fiberglass, suggesting that NCL moderates the cascade of downstream events initiated by the presence of asbestos fibers. DNA degradation is typical in cells exposed to asbestos fibers,^31^ and this is a first instance of inhibition of the degradation process by the co‐presence of clinoptilolite.

**Figure 4:**
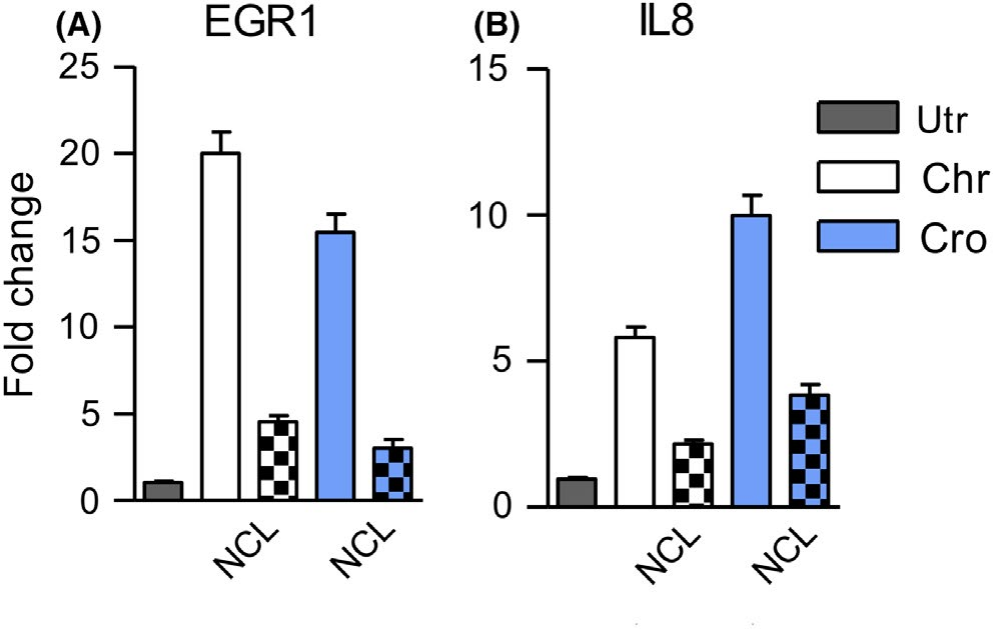
Mean expression levels of EGR1 and IL‐8, measured by RT‐qPCR, from cells treated with chrysotile (n = 12) and crocidolite (n = 4 assays), with or without natural clinoptilolite (checked bars) for 48 h. For all in vitro assays, asbestos was used at 5 µg/cm^2^ and zeolites at 10 µg/cm^2^

Crocidolite is known for its capacity to significantly alter the A549 transcriptome,^27^ affecting a multiplicity of functions including apoptosis and cell proliferation. We selected IL‐8 and EGR1 from this dataset because their expression levels were among the most highly up‐regulated. We found that they were overexpressed five to 20‐fold in A549 cells exposed to chrysotile or crocidolite but remained nearer control levels when NCL was also present (Fig. 4). The asbestos‐induced increase in expression of IL‐8 and EGR1 seen here were similar to levels seen in the full transcriptome study.^27^ The characterization of the genomic and epigenetic landscape of mesothelioma using diverse high‐throughput technologies has been reviewed recently.^34^ It identified a vast spectrum of coding and non‐coding transcriptome changes driving mesothelioma development, but only a fraction of this landscape has been identified by specific mechanistic changes and several of these are common to human cancer development.

In all these in vitro experiments, the commensurate presence of natural or synthetic zeolites alleviated asbestos toxicity similarly in two cell lines—A549 and MeT‐5A—of different origin. To explore this further, we envisage additional in vitro experiments using mesothelioma specific cell lines.

### 3.2 Zeolites moderate the effects of asbestos fibers in a mouse model of MM

**Figure 5:**
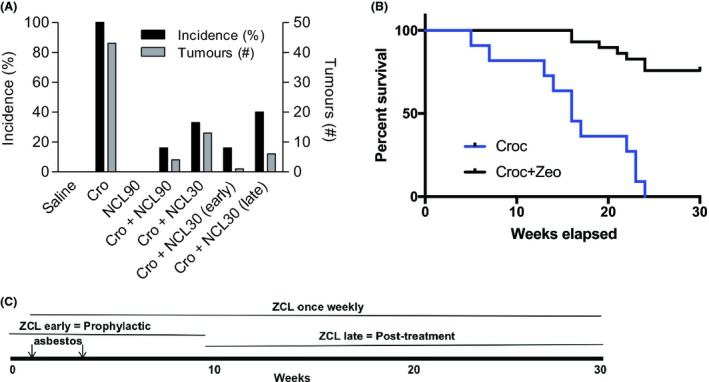
A, Zeolites reduce asbestos‐induced mesothelioma formation in a mouse model. Mice were treated with crocidolite, NCL or a combination, and monitored for 30 wk. NCL30 and NCL90 are natural clinoptilolite at 30 and 90 mg/kg, respectively. Early NCL represents the subgroup of mice that were given weekly NCL injections until week 10, with late NCL the subgroup receiving NCL injections from week 10. One mouse in the late subgroup developed a foot infection and was culled at week 8. Incidence (*y*‐axis) represents the onset of mesothelioma. B, Kaplan Meier plots of surviving mice treated with crocidolite only (Croc; blue trace), or crocidolite plus clinoptilolite (Croc + Zeo; black trace). C, The timeline of treatments over the 30 wk of the trials are given here. Crocidolite was injected in weeks 1 and 4 only. Clinoptilolite was injected weekly in Trial 1, or for the first 10 wk or last 20 wk in Trial 2. KM plots are not shown for saline or clinoptilolite controls as all of these mice survived the 30 wk of the trials. Complete post‐mortem data are given in Tables S1 and S2

The marked protection against asbestos toxicity afforded by zeolites in vitro led us to investigate their effects in an animal model of MM. After injection with crocidolite, all 13 MexTAg299 mice developed tumors in the abdominal cavity (Fig. 5), consistent with the first description of this model.^20^ In contrast, only five of the 18 crocidolite plus NCL‐treated mice developed tumors before the end of the trial at week 30. The early and late NCL subgroups had 1 and 2 mice with tumors, respectively, of 11 mice in total for these subgroups. There were no MM tumors in the control saline or NCL mouse subgroups of three mice each, and prolonged NCL injections were well tolerated (Tables S1 and S2 for complete post‐mortem results). Ascites fluid, occasionally bloody, was present in all asbestos‐treated mice, but not in the zeolite group. Interestingly, large volumes of ascitic fluid are often found in humans with peritoneal MM.^35^ The combined results in Fig. 5A are depicted as Kaplan Meier plots (Fig. 5B), which track the surviving treated mice over the 30 weeks of the trials. The injection protocol is given in Fig. 5C. These results indicated that NCL was able to improve survival whether given early or late in disease development. The significant survival numbers for late NCL injections, when tumors were almost certainly initiated, augurs well for a more expansive study of delayed clinoptilolite treatments, in order to establish the translational potential of treatment regimens.

Figure S2 compares abdominal cavities of mice representing control saline or clinoptilolite mice, and mice treated with crocidolite or crocodolite plus clinoptilolite. Post‐mortems such as these revealed pinpoint tumors of 1‐3 mm diameter (Fig. S2iv), and swollen liver, dilated cecum, spleen enlargement, and organ adhesions. Except for occasional clinoptilolite deposits (Fig. S2ii), 21/29 asbestos plus clinoptilolite mice were normal to the naked eye. Epithelioid and sarcomatoid tumor histologies are shown in Fig. S3.

**Figure 6:**
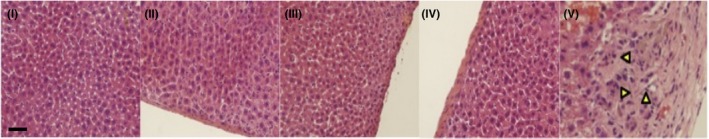
Sagittal liver sections from mice treated with: (i) saline; (ii) NCL30; (iii) NCL90; (iv) crocidolite plus NCL30; (v) crocidolite. In the last panel three asbestos fibers are arrowed. Saline and NCL‐treated control mice showed normal peritoneal lining of liver cells under HE staining, as did liver cells from mice injected with crocidolite plus NCL, whereas mice injected with crocidolite developed thickened peritoneum, liver swelling, and disorganisation. This was reflected in the tumor histology with a total of 14 mesothelial tumors among the five mice. The scale bar is 75 µm

The few mice in the crocidolite + NCL subgroup that developed tumors showed identical liver histology to the mice injected with crocidolite alone (Fig. 6i‐v), but the liver histology of all other crocidolite + NCL mice was identical to that of the saline and NCL controls (Fig. 6i‐iv).

**Figure 7:**
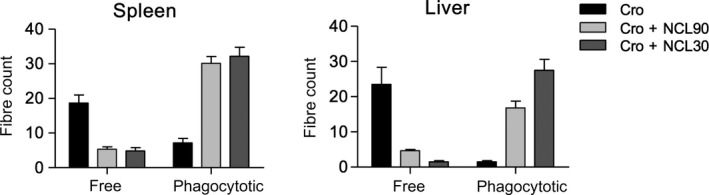
Total counts of free or phagocytosed asbestos fibers in liver and spleen sections. For each organ, sections (10 fields at 40×) from 6 asbestos and 12 asbestos plus NCL‐treated mice, were counted

There was clear evidence of free and phagocytosed crocidolite fibers in spleen and liver (Fig. S4; Fig. 7). A total of 42 free and 7 phagocytosed crocidolite fibers were counted in sections from asbestos‐injected mice taken at random from subgroups in both trials. In contrast, 12 free and 107 phagocytosed fibers were counted in spleen and liver sections from asbestos plus NCL mice (Fig. 7). Protection against asbestos carcinogenesis and increased survival was observed not only in all mice receiving weekly NCL for the entire 30‐week trial, but also when zeolites were given only for the first 10 weeks, or from week 10 onwards.

**Figure 8:**
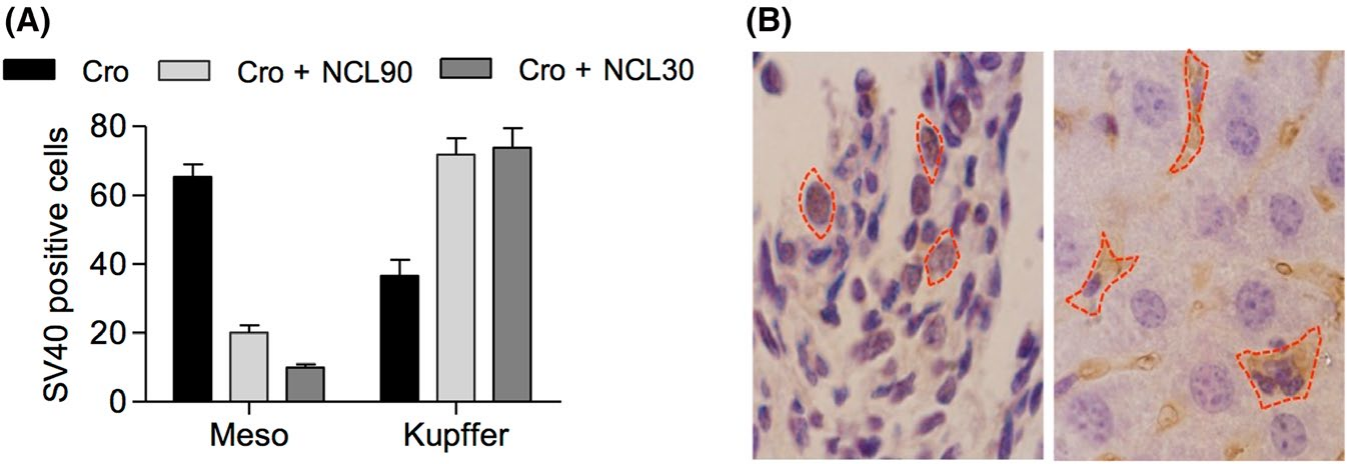
A, Total counts of SV40‐positive mesothelial cells (first three bars), or SV40‐negative Kupffer cells (right three bars), from asbestos‐treated mice (black), asbestos plus NCL90 mice (light gray), and asbestos plus NCL30 mice (dark gray). Ten fields at 40× were counted, covering 80%‐100% of each slide. Asbestos‐treated mice showed higher numbers of SV40 positive mesothelial cells (65) than did asbestos plus NCL mice (29); and correspondingly lower numbers of SV40‐negative Kupffer cells (38 *vs* 152) for asbestos *vs* asbestos plus NCL mice. B, SV40 immunohistochemistry on sections of mesotheliomas from an asbestos‐treated mouse (left panel), and liver tissue from an asbestos plus NCL‐treated mouse (right). The dashed red lines depict SV40 antigen within the mesothelial nuclei (left), or blue counterstained Kupffer cell nuclei (right). SV40 antigen staining is more obvious in the Kupffer macrophages than in the mesothelial cells due to the much larger size of the former cells. The scale bar is 40 µm

The expression of SV40 TAg indicates the effect of crocidolite asbestos in transforming mesothelial cells. There was a marked difference in the SV40‐positive mesothelial and SV40 negative Kupffer cells in mice treated with crocidolite alone or with crocidolite plus clinoptilolite (Fig. 8A). TAg was seen mainly in the nuclei of tumor cells (Fig. 8B; left histology panel). The reduced expression of SV40 TAg in crocidolite plus clinoptilolite‐treated mice might be caused by the amelioration of mesothelial cell transformation and thus a reduction in the initiation of mesothelioma. This study also detected the SV40 TAg inside Kupffer cells in the liver. Asbestos fibers were able to cause inflammation in both peritoneum and liver, but the Kupffer cells can only respond to the inflammation inside the liver. After asbestos plus clinoptilolite treatment, TAg was seen mainly in the cytoplasm of some Kupffer cells in liver tissue (Fig. 8B; right panel), most likely due to phagocytosis of SV40 positive mesothelial cells, which is consistent with a similar observation in the original description of this mouse model.^20^ There was no TAg expression in either saline or NCL‐treated mice (not shown). A thickened peritoneum covering the liver, indicative of an inflammatory reaction, was present in all asbestos‐treated mice. No such liver peritoneal swelling occurred in surviving asbestos plus NCL injected mice. The five asbestos plus NCL‐treated mice that developed tumors showed liver peritoneal thickening, but it was less evident than that seen in asbestos‐treated mice.

## 4 DISCUSSION

Our studies in vitro*,* and in an asbestos‐induced MM mouse model in vivo*,* support the results of previous studies demonstrating the ability of zeolites to prevent or delay carcinogenesis, but this is the first report of success in delaying or preventing MM in an animal model. In mice and dogs suffering from a variety of tumor types, zeolite injections (i.p.) led to improvements in overall health, life span, and decreases in tumor size.^36^ Micronized zeolite administered by gastric intubation to mice injected with melanoma cells significantly reduced the number of melanoma metastases,^36^ which suggests that zeolites might attenuate survival signals and induce tumor suppressor genes, notions that may be relevant to this study of asbestos‐induced MM. Sodium aluminosilicates, of which NCL is a derivative, are well tolerated and generally regarded as safe, and carcinogenic effects have not been observed. In one dietary study in humans, NCL promoted the excretion of heavy metals in urine without removing vital electrolytes, and only very high doses (300 mg/kg) caused a focal storage type reaction in lung tissue.^37^

The apparent safety of zeolites compared with asbestos, despite both being silicate minerals, may be related to their physical structure. Whereas asbestos is a fibrous material, most zeolites have orthorhombic crystalline structures with accessible open channels for water and large ions.^21^, ^22^ The safety of zeolites and, in particular, clinoptilolite, has been tested in animals and humans, by ingestion or injection.^36^ The European Food Safety Authority panel on additives in animal feed (2013) reported that clinoptilolite was non‐toxic at doses of 10 000 mg/kg.^22^ An earlier comprehensive toxicology study 38 used micronized clinoptilolite as a dietary supplement in rats and mice for up to one year, finding that doses of 1000 mg/mice/day elicited no apparent side effects or toxicity. In contrast, it is well known that the rod‐shaped or serpentine nature of asbestos fibers contributes to their toxicity.^33^ It is apposite that the only known carcinogenic zeolite, namely erionite, also has a rod‐like structure,^23^, ^24^, ^25^ which is in contrast to the cage‐like geometries of other zeolites.^21^, ^26^ A recent in vitro study showed that extremely small carbon nanotubes, as 10‐20 µm rod‐shaped particles, suppress normal immune responses in human lung epithelial cells,^39^, ^40^ or induce MM when administered to mice,^40^ mimicking what is seen with asbestos or erionite fibers. Interestingly, fullerenes do not generate the effects seen with nanocarbon rod‐shaped nanotubes, presumably because fullerenes have spherical/ellipsoidal structure.^41^ Synthetic chrysotile‐like nanofibers, devoid of iron, did not elicit oxidative stress, nor exert genotoxic and cytotoxic effects in a murine alveolar macrophage cell line, whereas the same nanofibers, loaded with 0.94% (w/w) iron, induced DNA strand breaks, lipoperoxidation, and oxidative stress, similarly to natural chrysotile.^42^

In support of the iron sequestering capacity of zeolites, our earlier study in MexTAg299 mice showed that clinoptilolite prevented abdominal organ discoloration and cell damage caused by iron polymaltose injected i.p. twice weekly for 30 weeks.^43^ There is a link between the immune system and iron homeostasis, chiefly via interactions between the iron regulator hepcidin, storage protein ferritin, and iron exporter ferroportin.^44^ Hepcidin is produced in the liver by a variety of immune cells, including macrophages and lymphocytes.^45^ We see a link between these findings and exposure to asbestos fibers, which leads to the suppression of the immune system and the generation of ROS, chiefly from labile Fe^3+^. In these circumstances, hepcidin is less able to remove iron. Mouse peritoneal macrophages exposed to Fe^3+^ in culture showed a marked decline in survival rate and an enhanced capacity for lipid peroxidation.^46^ The electrical activity of macrophages may be compromised by the presence of Fe^3+^ derived from aberrant metabolic function or foreign sources such as asbestos, and this probably extends to the immune system generally. By restoring iron homeostasis, zeolites may stimulate the immune system.^22^

The immunostimulatory effects of zeolites are well documented 36, 47, 48; and it is interesting to speculate on the interaction between zeolites, macrophages, and iron, given that there is a delicate balance of macrophages and free iron in mammals. Mouse peritoneal macrophages exposed to Fe^3+^ in culture showed a pronounced decline in survival.^46^ Mice injected intraperitoneally with clinoptilolite showed an increase in the number of peritoneal macrophages,^36^ which the authors ascribed to increased activation of macrophages and stimulants of the immune system. In our earlier iron mouse model study,^43^ we showed that the number of iron‐containing liver macrophages in iron plus clinoptilolite‐treated mice was double that seen in iron‐only treated mice. Interestingly, turning to other models of cancer, it has been shown that PD‐1 expression correlates negatively with phagocytic potency against tumor cells, and blockade of the PD‐1/PD‐L1 in vivo increases phagocytosis by macrophages, reduces tumor growth and lengthens the survival of mice in mouse models of cancer in a macrophage‐dependent fashion.^49^ From the viewpoint of the present study, we would like to explore the density and polarity of tumor‐infiltrating macrophages relative to the treatment states. Factors underlying the macrophage activation should also be studied. For example, are they interferon‐gamma dependent?

The continuing slow progress in identifying new treatments for MM and asbestosis is a factor that underlines the potential of this study and its outcomes. Whilst animal studies are notoriously difficult to reproduce, we have demonstrated similar statistically significant mouse survival rates in both of our pilot studies. Nevertheless, we acknowledge that this represents preliminary data and a better understanding of the mechanisms underlying the modulating effects of clinoptilolite is needed. It would be of interest to know if the ROS induced by asbestos exposure is mutagenic and if so, whether the mutation burden is reduced by the presence of zeolite. This would be a difficult task to prove, as asbestos is not considered very mutagenic and the mutational burden in mesothelioma is low 50, 51 but in‐depth transcriptomic and epigenomic analyses may reveal insights. Further research, including animal toxicology studies of clinoptilolite and trials with larger numbers of mice, will be required before any potential clinical studies are considered.

This pilot study suggests that zeolites are capable of ameliorating or delaying asbestos‐induced damage. In vivo this appears to involve reversing macrophage suppression, thus enabling macrophages to clear asbestos fibers and iron by phagocytosis and to scavenge free or asbestos‐adsorbed iron, thereby reducing ROS production. Our results support previous studies suggesting that chelation and/or prevention of redox imbalance may be a way to ameliorate asbestos toxicity,^17^, ^52^ but much more work is needed. While the notion of using one type of silicate mineral (zeolite) to counteract the deleterious effects of another (asbestos) is seemingly antithetical, the effects of zeolites demonstrated here point toward a potential means to reduce asbestos‐induced carcinogenesis. At this point, however, it is too early to speculate on the potential clinical application of our findings. Carefully planned additional studies to understand the mechanisms underlying the asbestos‐ameliorating effects of zeolites are required before any clinical testing can be considered. More importantly, such studies have the potential to shed further light on asbestos carcinogenesis and the role of the immune system in the development of mesothelioma, and may uncover new treatment targets.

## CONFLICT OF INTEREST

The authors declare no competing financial interests.

## AUTHOR CONTRIBUTIONS

The work was planned by AMG, with contributions from G.R., J.R., X.F., K.S., and C.M. The experiments were performed by X.F., C.M., K.S., T.J., and A.M.G. The manuscript was written by A.M.G., K.S., and G.R. Intellectual input was provided by J.R., G.R., C.R., K.S., and T.J.

## NOVELTY STATEMENT

Asbestos inhalation causes malignant mesothelioma (MM) and lung cancer, essentially incurable diseases with few effective treatments and dismal prognosis. We investigated the ability of the zeolite clinoptilolite to counteract the effects of asbestos in human cell lines and an asbestos‐induced mouse model of MM. Clinoptilolite prevented asbestos‐induced cell death, ROS production and DNA degradation in vitro, and significantly reduced MM development in vivo. Further exploration of the chemopreventive potential of zeolites is warranted.

## References

[1] Roe OD , Stella GM . Malignant pleural mesothelioma: history, controversy and future of a manmade epidemic. Eur Respir Rev. 2015;24:115‐131. 25726562 10.1183/09059180.00007014 PMC9487774

[2] Nagai H , Ishihara T , Lee WH , et al. Asbestos surface provides a niche for oxidative modification. Cancer Sci. 2011;102:2118‐2125. 21895868 10.1111/j.1349-7006.2011.02087.x PMC11158102

[3] Doll R . Mortality from lung cancer in asbestos workers. Br J Ind Med. 1955;12:81‐86. 14363586 10.1136/oem.12.2.81 PMC1037613

[4] Roggli VL , Vollmer RT . Twenty‐five years of fiber analysis: what have we learned? Hum Pathol. 2008;39:307‐315. 18187182 10.1016/j.humpath.2007.07.005

[5] Baldys A , Aust AE . Role of iron in inactivation of epidermal growth factor receptor after asbestos treatment of human lung and pleural target cells. Am J Respir Cell Mol Biol. 2005;32:436‐442. 15626777 10.1165/rcmb.2004-0133OC

[6] Robinson BW , Lake RA . Advances in malignant mesothelioma. New Engl J Med. 2005;353:1591‐1603. 16221782 10.1056/NEJMra050152

[7] Mossman BT , Shukla A , Heintz NH , Verschraegen CF , Thomas A , Hassan R . New insights into understanding the mechanisms, pathogenesis, and management of malignant mesotheliomas. Am J Pathol. 2013;182:1065‐1077. 23395095 10.1016/j.ajpath.2012.12.028 PMC3657618

[8] Cleaver AL , Bhamidipaty K , Wylie B , et al. Long‐term exposure of mesothelial cells to SV40 and asbestos leads to malignant transformation and chemotherapy resistance. Carcinogenesis. 2014;35:407‐414. 24130165 10.1093/carcin/bgt322

[9] Thapa B , Watkins DN , John T . Immunotherapy for malignant mesothelioma: reality check. Expert Rev Anticancer Ther. 2016;16:1‐10. 10.1080/14737140.2016.1241149 27669108

[10] Hardy JA , Aust AE . The effect of iron binding on the ability of crocidolite asbestos to catalyze DNA single‐strand breaks. Carcinogenesis. 1995;16:319‐325. 7859364 10.1093/carcin/16.2.319

[11] Fubini B , Barcelo F , Otero Arean C . Ferritin adsorption on amosite fibers: possible implications in the formation and toxicity of asbestos bodies. J Toxicol Environ Health. 1997;52:343‐352. 9354179 10.1080/00984109708984069

[12] Nishimura Y , Maeda M , Kumagai‐Takei N , et al. Altered functions of alveolar macrophages and NK cells involved in asbestos‐related diseases. Environ Health Prev Med. 2013;18:198‐204. 23463177 10.1007/s12199-013-0333-y PMC3650181

[13] Merchant JA . Human epidemiology: a review of fiber type and characteristics in the development of malignant and nonmalignant disease. Environ Health Perspect. 1990;88:287‐293. 2272325 10.1289/ehp.9088287 PMC1567998

[14] Kamp DW , Graceffa P , Pryor WA , Weitzman SA . The role of free radicals in asbestos‐induced diseases. Free Radic Biol Med. 1992;12:293‐315. 1577332 10.1016/0891-5849(92)90117-y

[15] Hardy JA , Aust AE . Iron in asbestos chemistry and carcinogenicity. Chem Rev. 1995;95:97‐118.

[16] Kahlos K , Pitkanen S , Hassinen I , Linnainmaa K , Kinnula VL . Generation of reactive oxygen species by human mesothelioma cells. Br J Cancer. 1999;80:25‐31. 10.1038/sj.bjc.6690316 PMC2363004 10389973

[17] Kell DB . Iron behaving badly: inappropriate iron chelation as a major contributor to the aetiology of vascular and other progressive inflammatory and degenerative diseases. BMC Med Genomics. 2009;2:2. 19133145 10.1186/1755-8794-2-2 PMC2672098

[18] Benedetti S , Nuvoli B , Catalani S , Galati R . Reactive oxygen species a double‐edged sword for mesothelioma. Oncotarget. 2015;6:16848‐16865. 26078352 10.18632/oncotarget.4253 PMC4627278

[19] Robinson C , van Bruggen I , Segal A , et al. A novel SV40 TAg transgenic model of asbestos‐induced mesothelioma: malignant transformation is dose dependent. Can Res. 2006;66:10786‐10794. 10.1158/0008-5472.CAN-05-4668 17108115

[20] Robinson C , Walsh A , Larma I , O'Halloran S , Nowak AK , Lake RA . MexTAg mice exposed to asbestos develop cancer that faithfully replicates key features of the pathogenesis of human mesothelioma. Eur J Cancer. 2011;47:151‐161. 20850297 10.1016/j.ejca.2010.08.015

[21] Breck DW . Crystalline molecular sieves. J Chem Educ. 1964;41:678‐689.

[22] Kraljevic Pavelic S , Simovic Medica J , Gumbarevic D , Filosevic A , Przulj N , Pavelic K . Critical review on zeolite clinoptilolite safety and medical applications in vivo. Front Pharmacol. 2018;9:1350. 30538633 10.3389/fphar.2018.01350 PMC6277462

[23] Suzuki Y , Kohyama N . Malignant mesothelioma induced by asbestos and zeolite in the mouse peritoneal cavity. Environ Res. 1984;35:277‐292. 6092048 10.1016/0013-9351(84)90136-1

[24] Fach E , Waldman WJ , Williams M , Long J , Meister RK , Dutta PK . Analysis of the biological and chemical reactivity of zeolite‐based aluminosilicate fibers and particulates. Environ Health Perspect. 2002;110:1087‐1096. 12417479 10.1289/ehp.021101087 PMC1241064

[25] Gulumian M . An update on the detoxification processes for silica particles and asbestos fibers: successess and limitations. J Toxicol Environ Health. 2005;8:453‐483. 10.1080/10937400590952547 16188731

[26] Adamis Z , Tatrai E , Honma K , Six E , Ungvary G . In vitro and in vivo tests for determination of the pathogenicity of quartz, diatomaceous earth, mordenite and clinoptilolite. Ann Occup Hyg. 2000;44:67‐74. 10689760

[27] Hevel JM , Olson‐Buelow LC , Ganesan B , Stevens JR , Hardman JP , Aust AE . Novel functional view of the crocidolite asbestos‐treated A549 human lung epithelial transcriptome reveals an intricate network of pathways with opposing functions. BMC Genom. 2008;9:376. 10.1186/1471-2164-9-376 PMC2533023 18687144

[28] Brousset P , de Araujo V , Gascoyne RD . Immunohistochemical investigation of SV40 large T antigen in Hodgkin and non‐Hodgkin's lymphoma. Int J Cancer. 2004;112:533‐535. 15382082 10.1002/ijc.20397

[29] Goodwin EM , Zhong Q , Abendroth CS , Ward‐Kavanagh LK , Schell TD , Cooper TK . Anaplastic renal carcinoma expressing SV40 T antigen in a female TRAMP mouse. Comp Med. 2013;63:338‐341. 24209969 PMC3750669

[30] Fan X , Heijnen CJ , van der Kooij MA , Groenendaal F , van Bel F . Beneficial effect of erythropoietin on sensorimotor function and white matter after hypoxia‐ischemia in neonatal mice. Pediatr Res. 2011;69:56‐61. 20856165 10.1203/PDR.0b013e3181fcbef3

[31] Aljandali A , Pollack H , Yeldandi A , Li Y , Weitzman SA , Kamp DW . Asbestos causes apoptosis in alveolar epithelial cells: role of iron‐induced free radicals. J Lab Clin Med. 2001;137:330‐339. 11329530 10.1067/mlc.2001.114826

[32] Broaddus VC , Yang L , Scavo LM , Ernst JD , Boylan AM . Asbestos induces apoptosis of human and rabbit pleural mesothelial cells via reactive oxygen species. J Clin Investig. 1996;98:2050‐2059. 8903324 10.1172/JCI119010 PMC507649

[33] Rascoe PA , Jupiter D , Cao X , Littlejohn JE , Smythe WR . Molecular pathogenesis of malignant mesothelioma. Expert Rev Mol Med. 2012;14:e12. 22622048 10.1017/erm.2012.6

[34] Sage AP , Martinez VD , Minatel BC , et al. Genomics and epigenetics of malignant mesothelioma. High Throughput. 2018;7(3):20. 10.3390/ht7030020 PMC6163664 30060501

[35] Munkholm‐Larsen S , Cao CQ , Yan TD . Malignant peritoneal mesothelioma. World J Gastrointest Surg. 2009;1:38‐48. 21160794 10.4240/wjgs.v1.i1.38 PMC2999110

[36] Pavelic K , Katic M , Sverko V , et al. Immunostimulatory effect of natural clinoptilolite as a possible mechanism of its antimetastatic ability. J Cancer Res Clin Oncol. 2002;128:37‐44. 11862470 10.1007/s00432-001-0301-6 PMC12164407

[37] Flowers JL , Lonky SA , Deitsch EJ . Clinical evidence supporting the use of an activated clinoptilolite suspension as an agent to increase urinary excretion of toxic heavy metals. Nutr Diet Suppl. 2009;1:11‐18.

[38] Pavelic K , Hadzija M , Bedrica L , et al. Natural zeolite clinoptilolite: new adjuvant in anticancer therapy. J Mol Med. 2001;78:708‐720. 11434724 10.1007/s001090000176

[39] Herzog E , Byrne HJ , Casey A , et al. SWCNT suppress inflammatory mediator responses in human lung epithelium in vitro. Toxicol Appl Pharmacol. 2009;234:378‐390. 19041333 10.1016/j.taap.2008.10.015

[40] Takagi A , Hirose A , Nishimura T , et al. Induction of mesothelioma in p53+/‐ mouse by intraperitoneal application of multi‐wall carbon nanotube. J Toxicol Sci. 2008;33:105‐116. 18303189 10.2131/jts.33.105

[41] Hirai T , Yoshioka Y , Udaka A , et al. Potential suppressive effects of two C60 fullerene derivatives on acquired immunity. Nanoscale Res Lett. 2016;11:449. 27709563 10.1186/s11671-016-1663-7 PMC5052157

[42] Gazzano E , Turci F , Foresti E , et al. Iron‐loaded synthetic chrysotile: a new model solid for studying the role of iron in asbestos toxicity. Chem Res Toxicol. 2007;20:380‐387. 17315889 10.1021/tx600354f

[43] Fan X , McLaughlin C , Ravasini J , Robinson C , George AM . Zeolite protects mice from iron‐induced damage in a mouse model trial. FEBS Open Bio. 2018;8:1773‐1781. 10.1002/2211-5463.12477 PMC6212648 30410857

[44] Cherayil BJ . Iron and immunity: immunological consequences of iron deficiency and overload. Arch Immunol Ther Exp (Warsz). 2010;58:407‐415. 20878249 10.1007/s00005-010-0095-9 PMC3173740

[45] Pinto JP , Dias V , Zoller H , et al. Hepcidin messenger RNA expression in human lymphocytes. Immunology. 2010;130:217‐230. 20102409 10.1111/j.1365-2567.2009.03226.x PMC2878466

[46] Abok K , Hirth T , Ericsson JL , Brunk U . Effect of iron on the stability of macrophage lysosomes. Virchows Arch B Cell Pathol Incl Mol Pathol. 1983;43:85‐101. 6137109 10.1007/BF02932947

[47] Ivkovic S , Deutsch U , Silberbach A , Walraph E , Mannel M . Dietary supplementation with the tribomechanically activated zeolite clinoptilolite in immunodeficiency: effects on the immune system. Adv Ther. 2004;21:135‐147. 15310086 10.1007/BF02850340

[48] Martin‐Kleiner I , Flegar‐Mestric Z , Zadro R , et al. The effect of the zeolite clinoptilolite on serum chemistry and hematopoiesis in mice. Food Chem Toxicol. 2001;39:717‐727. 11397518 10.1016/s0278-6915(01)00004-7

[49] Gordon SR , Maute RL , Dulken BW , et al. PD‐1 expression by tumour‐associated macrophages inhibits phagocytosis and tumour immunity. Nature. 2017;545:495‐499. 28514441 10.1038/nature22396 PMC5931375

[50] Bueno R , Stawiski EW , Goldstein LD , et al. Comprehensive genomic analysis of malignant pleural mesothelioma identifies recurrent mutations, gene fusions and splicing alterations. Nat Genet. 2016;48:407‐416. 26928227 10.1038/ng.3520

[51] Hmeljak J , Sanchez‐Vega F , Hoadley KA , et al. Integrative molecular characterization of malignant pleural mesothelioma. Cancer Discov. 2018;8:1548‐1565. 30322867 10.1158/2159-8290.CD-18-0804 PMC6310008

[52] Poggiali E , Cassinerio E , Zanaboni L , Cappellini MD . An update on iron chelation therapy. Blood Transfus. 2012;10:411‐422. 22790257 10.2450/2012.0008-12 PMC3496216

