# Supplementary material for: Zeolites ameliorate asbestos toxicity in a transgenic model of malignant mesothelioma
Source: FASEB Bioadv. 2019 Aug 22;1(9):550–60. doi: 10.1096/fba.2019-00040 (PMC6996371; doi:10.1096/fba.2019-00040)

Supplementary Figure 1

Viability assays for zeolite-treated A549 (left) and MeT-5A (right) cells conducted under the same conditions as in 'Methods' for Figure 1a. Viability was adjusted to 100% for untreated cells and the percentage viabilities calculated for the treatments with natural clinoptilolite (NCL), synthetic clinoptilolite (SCL), and synthetic mordenite (SMO). The results show that the three zeolites are relatively non-toxic in terms of cell viability at concentrations up to ten times those used in the asbestos-treated cells assays. Both A549 and Met-5A cell types survived to about 90% of the viability of untreated cells at 5 times the zeolite concentrations (50 μg/cm^2) used in this study, and at 97-98% compared to untreated cell survival at the concentration (10 μg/cm^2) used in this study. The relatively innocuous effects of high concentrations of zeolites on A549 and Met-5A cell viability though pertinent for this study, might not be the case for example in primary lung cells.

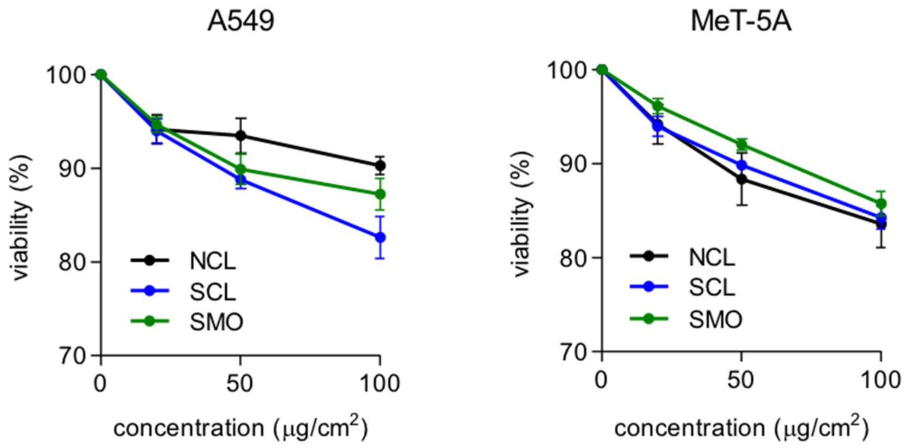

Supplementary Figure 2

Abdominal cavities of mice post-mortem. (i) Saline treated mouse, with normal appearance of tissues and organs; (ii) NCL-treated mouse, with clinoptilolite deposits arrowed (removed and examined by microscopy) in an otherwise normal abdomen; (iii) Asbestos plus clinoptilolite –treated mouse, with clinoptilolite deposit arrowed in an otherwise normal abdomen (iv) Asbestos-treated mouse with tumours indicated by arrows. ~6 mL of Ascites fluid was taken from this mouse (inset), which reached a humane endpoint at week 16.

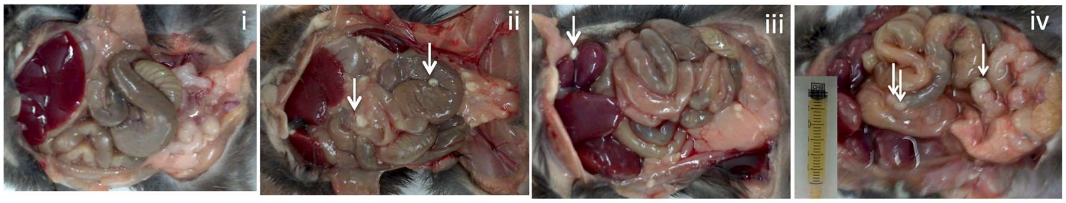

Supplementary Figure 3

Multiple tumours resembling epithelioid (i), and sarcomatoid (ii) mesotheliomas were detected in the peritoneum of crocidolite-injected mice. The scale bar is 50 μm.

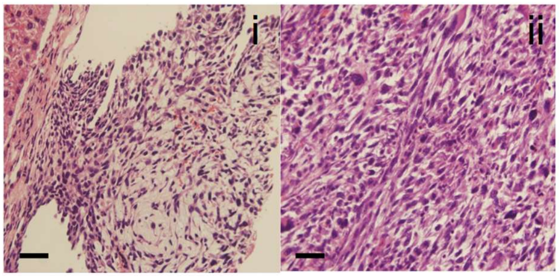

Supplementary Figure 4

Sample H&E and Prussian blue stained liver sections from mice treated with crocidolite (top panels) or crocidolite+NCL30 (bottom panels). Asbestos fibres are depicted within the rectangles. Very few fibres were found in the crocidolite+NCL30 treated cells, e.g. single rectangle (bottom right panel), but there were macrophages present in most of these sections (circles). These sections are typical of the many sections taken from tissues. The scale bar is 25 μm.

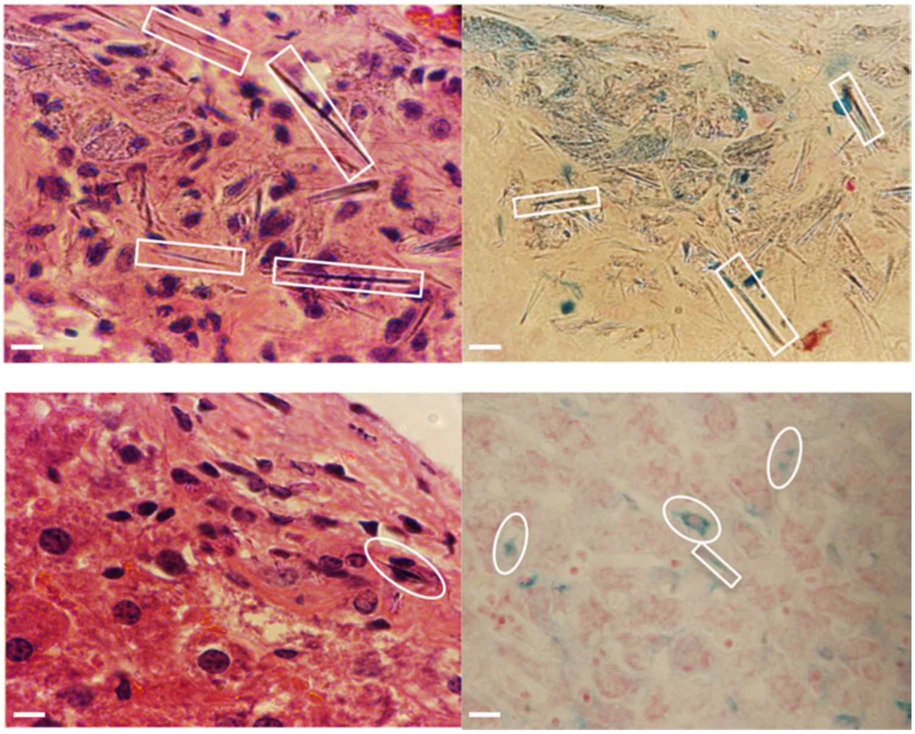

Supplement: Supplementary file 1 [file FBA2-1-550-s001.pdf]
