# Supplementary material for: Zeolites ameliorate asbestos toxicity in a transgenic model of malignant mesothelioma
Source: FASEB Bioadv. 2019 Aug 22;1(9):550–60. doi: 10.1096/fba.2019-00040 (PMC6996371; doi:10.1096/fba.2019-00040)
Supplement: Supplementary file 2 [file FBA2-1-550-s002.pdf]

Supplementary Table 1. Post-mortem summary for the mice in Trial 1.

Mouse ID	Gender	Treatment	PM (wk)	Liver	Spleen	Kidney	Abdomen	Ascites (mL)
xc01	M	Saline	30	N				
xc02	M	Saline	30	N				
xc03	F	Saline	30	N				
xc04	F	NCL	30		Slightly swollen			
xc05	F	NCL	30					
xc06	M	NCL	30					
xc07	M	Crocidolite	23	Swollen	Adhered to stomach		2x2mm tumours	1.5
xc08	M	Crocidolite	22	Swollen	Adhered to stomach		Dilated caecum; 2x3mm tumours	3
xc09	M	Crocidolite	17	Swollen	Adhered to stomach		3x1mm, 1x2cm tumours;	
xc10	F	Crocidolite	16	Swollen	Adhered to stomach; pancreas	1x1mm tumour	5x1mm tumours;	6
xc11	F	Crocidolite	25	Swollen	Enlarged; adhered stomach		2x2mm tumours	1
xc12	F	Crocidolite	16	Swollen; adhered to stomach	Enlarged; adhered stomach	Organ pale; 1 x 2 mm tumour	2x1mm tumours	1
xd01	M	Crocidolite + NCL90	30					0.2
xd02	M	Crocidolite + NCL90	24	Swollen; 1x1mm tumour	Adhered to stomach	1x1mm tumour	2x1mm tumours	4
xd03	M	Crocidolite + NCL90	30	Slightly swollen	Slightly enlarged			
xd04	F	Crocidolite + NCL90	30	Slightly swollen	Slightly enlarged			
xd05	F	Crocidolite + NCL90	30	Slightly swollen	Adhered to stomach			
xd06	F	Crocidolite + NCL90	30					
xd07	M	Crocidolite + NCL30	30	Slightly swollen				
xd08	M	Crocidolite + NCL30	30	Swollen; 1x1mm tumour		3x1mm tumours	1x2mm tumour	5
xd09	M	Crocidolite + NCL30	16	Slightly swollen	Adhered to gut			
xd10	F	Crocidolite + NCL30	30					
xd11	F	Crocidolite + NCL30	30	Slightly swollen	Slightly enlarged			0.3
xd12	F	Crocidolite + NCL30	30					
