# Supplementary material for: Zeolites ameliorate asbestos toxicity in a transgenic model of malignant mesothelioma
Source: FASEB Bioadv. 2019 Aug 22;1(9):550–60. doi: 10.1096/fba.2019-00040 (PMC6996371; doi:10.1096/fba.2019-00040)
Supplement: Supplementary file 3 [file FBA2-1-550-s003.pdf]

Supplementary Table 2. Post-mortem summary for the mice in Trial 2.

Mouse ID	Gender	Treatment	PM (wk)	Liver	Spleen	Kidney	Abdomen	Ascites (mL)
xf01	F	Crocidolite	5	Pale; swollen; adhered to stomach	Enlarged; adhered to stomach	Pale	Adhered tissue	0.4
xf02	F	Crocidolite	24	Swollen; 2x1mm tumours	Adhered to stomach	1x5mm tumour	2x2mm tumours	0.2
xf03	F	Crocidolite	7	Adhered to diaphragm	Enlarged; adhered to stomach; 5x1mm tumours	Adhered to spleen	Caecum adhered to fallopian tubes; 1x1 mm tumour	1.5; bloody
xf04	M	Crocidolite	23	Swollen; adhered to gut	Adhered to stomach		1x1mm tumour; 3x2mm tumours	0.5
xf05	M	Crocidolite	14	Swollen	Enlarged; adhered to stomach		Enlarged caecum; 1x2mm tumour	0.2
xf06	M	Crocidolite	13	Swollen; 2x1mm tumours	Adhered to stomach	1x1mm tumour	2x3mm tumours; stomach adhered to spleen and liver	0.5
xf07	F	Crocidolite	24	Swollen; adhered to stomach	Enlarged; adhered to stomach; 3x1mm tumours			3.5
xf08	F	Crocidolite + Late NCL	30					
xf09	F	Crocidolite + Late NCL	24	Adhered to stomach; 1x2mm tumour	Enlarged; adhered to stomach; 2x1mm tumours		2x2mm tumours	2; bloody
xf10	M	Crocidolite + Late NCL	30					
xf11	M	Crocidolite + Late NCL	19	Slightly swollen	Adhered to stomach; 1x2mm tumour			1
xf12	M	Crocidolite + Late NCL	30		Slightly enlarged			0.2
xg01	F	Crocidolite + NCL	30					
xg02	F	Crocidolite + NCL	30	Slightly swollen				
xg03	F	Crocidolite + NCL	21	Swollen; Adhered to stomach	Enlarged; adhered to stomach	Adhered to spleen	2x2mm tumours	0.5
xg04	M	Crocidolite + NCL	30					
xg05	M	Crocidolite + NCL	30	Adhered to gut				
xg06	M	Crocidolite + NCL	22	Enlarged; 1x1mm tumour	Adhered to stomach	Adhered to liver; 2x1mm tumours	3x1mm tumours	3.5
xg07	F	Crocidolite + Early NCL	30					
xg08	F	Crocidolite + Early NCL	30					
xg09	F	Crocidolite + Early NCL	16	Slightly swollen	Enlarged; adhered to stomach		Enlarged caecum; 1x2mm tumour	
xg10	M	Crocidolite+ Early NCL	30					
xg11	M	Crocidolite + Early NCL	30		Adhered to stomach			
xg12	M	Crocidolite + Early NCL	30					
